# Optimal Translation Along a Circular mRNA

**DOI:** 10.1038/s41598-017-09602-6

**Published:** 2017-08-25

**Authors:** Yoram Zarai, Alexander Ovseevich, Michael Margaliot

**Affiliations:** 1 School of Electrical Engineering, Tel-Aviv University, Tel-Aviv, Israel; 2 Institute for Problems in Mechanics, Russian Academy of Sciences, Moscow, Russia; 3 School of Electrical Engineering, Tel-Aviv University, Tel-Aviv, Israel

## Abstract

The ribosome flow model on a ring (RFMR) is a deterministic model for ribosome flow along a circularized mRNA. We derive a new *spectral representation* for the optimal steady-state production rate and the corresponding optimal steady-state ribosomal density in the RFMR. This representation has several important advantages. First, it provides a simple and numerically stable algorithm for determining the optimal values even in very long rings. Second, it enables efficient computation of the sensitivity of the optimal production rate to small changes in the transition rates along the mRNA. Third, it implies that the optimal steady-state production rate is a strictly concave function of the transition rates. Maximizing the optimal steady-state production rate with respect to the rates under an affine constraint on the rates thus becomes a convex optimization problem that admits a unique solution. This solution can be determined numerically using highly efficient algorithms. This optimization problem is important, for example, when re-engineering heterologous genes in a host organism. We describe the implications of our results to this and other aspects of translation.

## Introduction

Gene expression is the process by which the information encoded in a gene is used to synthesize a functional gene product. Two main stages of this process are *transcription* in which the information in the DNA of a specific gene is copied into a messenger RNA (mRNA) molecule and *translation*. The latter includes three phases: (1) initiation: complex macro-molecules called ribosomes bind to the mRNA; (2) elongation: the ribosomes unidirectionally decode each codon into the corresponding amino-acid that is delivered to the awaiting ribosome by transfer RNA (tRNA) molecules; and (3) termination: the ribosome detaches from the mRNA, the amino-acid sequence is released, folded and becomes a functional protein^1^. The output rate of ribosomes from the mRNA, which is also the rate in which proteins are generated, is called the protein translation rate or production rate.

Translation occurs in all living organisms, and under almost all conditions, to generate the macromolecular machinery for life. Developing a deeper understanding of translation has important implications in numerous scientific disciplines including medicine, evolutionary biology, biotechnology, and synthetic biology. Computational models of translation are essential in order to better understand this complex, dynamical and tightly-regulated process. Such models can also aid in integrating and analyzing the rapidly increasing experimental findings related to translation (see, e.g refs 2–9).

Computational models of translation describe the dynamical flow of ribosomes along the mRNA molecule, and include parameters that encode the various factors affecting the codon decoding rates and the binding of ribosomes. Some of these models provide a framework for both rigorous analysis and Monte Carlo simulations, thus promoting a better understanding of the way the parameters, and other factors, affect the dynamical and steady-state behavior of translation. Several computational models have been suggested based on different paradigms for example kinetics-based ordinary differential equations (see, e.g ref. 10), Petri nets^11^, and probabilistic Boolean networks^12^. For more details, see the survey papers^9, 13^.

A standard mathematical model for ribosome flow is the *totally asymmetric simple exclusion process* (TASEP)^14, 15^. In this model, particles hop unidirectionally along an ordered lattice of *L* sites. Each site can be either free or occupied by a particle, and a particle can only hop to a free site. This *simple exclusion principle* models particles that have “volume” and thus cannot overtake one other. The hops are stochastic, and the rate of hoping from site *i* to site *i* + 1 is denoted by *γ*_*i*_. TASEP has two standard configurations. In TASEP with *open boundary conditions* the two sides of the lattice are connected to two particle reservoirs, and a particle can hop to [from] the first [last] site of the lattice at a rate *α* [*β*]. The average flow through the lattice converges to a steady-state value that depends on the parameters *α*, *γ*_1_, …, *γ*_*L*−1_, *β*. Analysis of TASEP with open boundary conditions is non trivial, and closed-form results have been obtained mainly for the homogeneous TASEP (HTASEP), i.e. for the case where all the *γ*_*i*_s are assumed to be equal.

In TASEP with *periodic boundary conditions* the chain is closed, and a particle that hops from the last site returns to the first one. Thus, here the lattice is a ring, and the total number of particles along the ring is conserved.

TASEP has become a fundamental model in non-equilibrium statistical mechanics, and has been applied to model numerous natural and artificial processes such as traffic flow, communication networks, and pedestrian dynamics^16^. In the context of translation, the lattice models the mRNA molecule, the particles are ribosomes, and simple exclusion means that a ribosome cannot overtake a ribosome in front of it.

**Figure 1:**
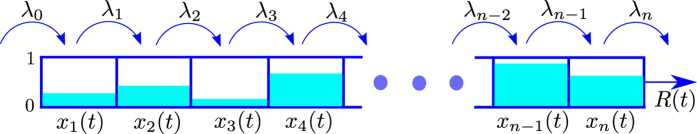
The RFM models unidirectional flow along a chain of *n* sites. The state variable *x*_*i*_(*t*) ∈ [0, 1] represents the density at site *i* at time *t*. The parameter *λ*_*i*_ > 0 controls the transition rate from site *i* to site *i* + 1, with *λ*_0_ > 0 [*λ*_*n*_ > 0] controlling the initiation [exit] rate. The output rate at time *t* is *R*(*t*) := *λ*_*n*_ *x*_*n*_(*t*).

The *ribosome flow model* (RFM)^17^ is a continuous-time deterministic model for the unidirectional flow of “material” along an open chain of *n* consecutive compartments (or sites). The RFM can be derived via a dynamic mean-field approximation of TASEP with open boundary conditions [16, section 4.9.7] [18, p. R345]. In a RFM with *n* sites, the state variable *x*_*i*_(*t*) ∈ [0, 1], *i* = 1, …, *n*, describes the normalized amount of “material” (or density) at site *i* at time *t*, where *x*_*i*_(*t*) = 1 [*x*_*i*_(*t*) = 0] indicates that site *i* is completely full [completely empty] at time *t*. In the RFM, the two sides of the chain are connected to two particle reservoirs. A parameter *λ*_*i*_ > 0, *i* = 0, …, *n*, controls the transition rate from site *i* to site *i* + 1, where *λ*_0_ [*λ*_*n*_] controls the initiation [exit] rate (see Fig. 1).

In the *ribosome flow model on a ring* (RFMR)^18, 19^ the particles exiting the last site reenter the first site. This is a dynamic mean-field approximation of TASEP with periodic boundary conditions. The RFMR admits a first integral, i.e. a quantity that is preserved along the dynamics, as the total amount of material is conserved. Both the RFM and RFMR are cooperative dynamical systems^20^, but their dynamical properties are quite different^19^.

Through simultaneous interactions with the cap-binding protein eIF4E and the poly(A)-binding protein PABP, the eukaryotic initiation factor eIF4G is able to bridge the two ends of the mRNA^21, 22^. This suggests that a large fraction of the ribosomes that complete translating the mRNA re-initiate. The RFMR is a good approximation of the translation dynamics in these circularized mRNAs. In addition, circular RNA forms (which includes covalent RNA interactions) appear in all domains of life^23–30^, and it was recently suggested that circular RNAs can be translated in eukaryotes^28–30^.

It was shown in ref. 19 that the RFMR admits a unique steady-state that depends on the initial total density along the ring and the transition rates, but not on the distribution of the total density among the sites. For a fixed set of transition rates, all trajectories emanating from initial conditions with the same total density converge to the unique steady-state. Ref. 31 considered the ribosomal density along a circular mRNA that *maximizes* the steady-state production rate using the RFMR. It was shown that given any arbitrary set of positive transition rates, there exists a *unique* optimal total density (the same is true for TASEP with periodic boundary condition^32^). However, this unique optimum was not given explicitly, other than under certain special symmetry conditions on the rates.

We note that the ribosomal density along the mRNA molecule plays a critical role in regulating gene expression, and specifically in determining protein production rates^33, 34^. For example, it was suggested in ref. 34 that the cell tightly regulates ribosomal densities in order to maintain protein concentrations at different growth temperatures. At higher temperatures, the ribosomal density along the mRNA “improves” in order to increase protein production rates (as protein stability decreases with temperature).

The ribosomal density also affects different fundamental intracellular phenomena. Traffic james, abortions, and collisions may form if the ribosomal density is very high^35^. It may also contribute to co-translational misfolding of proteins, which then requires additional resources in order to degrade the degenerated proteins^36–38^. On the other hand, a very low ribosomal density may lead to high degradation rate of mRNA molecules^39–42^. Thus, analyzing the ribosomal density that maximizes the production rate is critical in understanding how cells evolved to adapt and thrive in a changing environment.

Here we derive a new *spectral representation* (SR) for the optimal steady-state production rate and the corresponding steady-state ribosomal density in the RFMR. This SR has several important advantages. First, it provides a simple and numerically stable way to compute the optimal values even in very long rings. Second, it enables efficient computation of the sensitivity of the optimal steady-state production rate to small changes in the transition rates. This sensitivity analysis may find important applications in synthetic biology where a crucial problem is to determine the codons that are the most “important” in terms of their effect on the production rate. Also, sensitivity analysis is important because of the inherent stochasticity of the bio-molecular processes in the cell (see, e.g ref. 43).

Third, the SR implies that the optimal steady-state production rate is a strictly concave function of the transition rates. Thus, the problem of maximizing the optimal steady-state production rate *with respect to the rates* becomes a convex optimization problem that admits a unique solution. Furthermore, this solution can be determined numerically using highly efficient algorithms.

The remainder of this paper is organized as follows. The next two sub-sections briefly review the RFM and the RFMR. Section 2 describes our main results and their biological implications. Section 3 concludes and suggests several directions for further research. To increase the readability of this paper, the proofs of all the results are placed in the Appendix. We use standard notation. Vectors [matrices] are denoted by small [capital] letters. ${{\mathbb{R}}}^{n}$ is the set of vectors with *n* real coordinates. For a (column) vector $x\in {{\mathbb{R}}}^{n}$, *x*_*i*_ is the *i*th entry of *x*, and *x*′ is the transpose of *x*. Let ${{\mathbb{R}}}_{++}^{n}\,:=\{v\in {{\mathbb{R}}}^{n}:{v}_{i} > 0,i=1,\ldots ,n\}$, i.e. the set of all *n*-dimensional vectors with positive entries.

## Ribosome Flow Model (RFM)

In an RFM with *n* sites, the state variable *x*_*i*_(*t*) ∈ [0, 1], *i* = 1, …, *n*, denotes the density at site *i* at time *t*, where *x*_*i*_(*t*) = 1 [*x*_*i*_(*t*) = 0] means that site *i* is completely full [empty] at time *t*. The *n* + 1 parameters *λ*_*i*_ > 0, *i* = 0, …, *n*, control the transition rate from site *i* to site *i* + 1. The RFM is a set of *n* first-order nonlinear ordinary differential equations describing the change in the amount of “material” in each site:

1 $${\dot{x}}_{i}(t)={\lambda }_{i-1}{x}_{i-1}(t)(1-{x}_{i}(t))-{\lambda }_{i}{x}_{i}(t)(1-{x}_{i+1}(t)),\quad i=1,\ldots ,n,$$

where *x*_0_(*t*) := 1, *x*_*n*+1_(*t*) := 0, and ${\dot{x}}_{i}$ is the change in the amount of material at site *i* at time *t*, i.e. ${\dot{x}}_{i}(t)\,:=\frac{d}{dt}{x}_{i}(t)$, *i* = 1, …, *n*. Eq. (1) is a master equation: the change in density in site *i* is the flow from site *i* − 1 to site *i* minus the flow from site *i* to site *i* + 1. The first flow, that is, the input rate to site *i* is *λ*_*i*−1_ *x*_*i*−1_(*t*)(1 − *x*_*i*_(*t*)). This rate is proportional to *x*_*i*−1_(*t*), i.e. it increases with the density at site *i* − 1, and to (1 − *x*_*i*_(*t*)), i.e. it decreases as site *i* becomes fuller. In particular, when site *i* is completely full, i.e. when *x*_*i*_(*t*) = 1, there is no flow into this site. This is reminiscent of the simple exclusion principle: the flow of particles into a site decreases as that site becomes fuller. Note that the maximal possible flow from site *i* − 1 to site *i* is *λ*_*i*−1_. Similarly, the output rate from site *i*, which is also the input rate to site *i* + 1, is given by *λ*_*i*_ *x*_*i*_(*t*)(1 − *x*_*i*+1_(*t*)). The output rate from the chain is *R*(*t*) := *λ*_*n*_ *x*_*n*_(*t*), that is, the flow out of the last site.

In the context of translation, the *n*-sites chain is the mRNA, *x*_*i*_(*t*) describes the ribosomal density at site *i* at time *t*, and *R*(*t*) describes the rate at which ribosomes leave the mRNA, which is also the rate at which the proteins are generated. Thus, *R*(*t*) is the protein translation rate or production rate at time *t*.

Since every state-variable models the density of ribosomes in a site, normalized such that a value zero [one] corresponds to a completely empty [full] site, the state space of the RFM is the *n*-dimensional unit cube *C* ^*n*^ := [0, 1]^*n*^. Let *x*(*t*, *a*) denote the solution of the RFM at time *t* for the initial condition *x*(0) = *a*. It has been shown in ref. 44 (see also ref. 45) that for every *a* ∈ *C* ^*n*^ this solution remains in *C* ^*n*^ for all *t* ≥ 0, and that the RFM admits a globally asymptotically stable steady-state *e* ∈ int(*C* ^*n*^), i.e. ${\mathrm{lim}}_{t\to \infty }x(t,a)=e$ for all *a* ∈ *C* ^*n*^. The value *e* depends on the rates *λ*_0_, …, *λ*_*n*_, but not on the initial condition *x*(0) = *a*. This means that if we simulate the RFM starting from any initial density of ribosomes on the mRNA the dynamics will always converge to the same steady-state (i.e., to the same final ribosome density along the mRNA). In particular, the production rate *R*(*t*) = *λ*_*n*_ *x*_*n*_(*t*) always converges to the steady-state value:

2 $$R\,:={\lambda }_{n}{e}_{n}.$$

A spectral representation of this steady-state value has been derived in ref. 46. Given a RFM with dimension *n* and rates *λ*_0_, …, *λ*_*n*_, define a (*n* + 2) × (*n* + 2) Jacobi matrix

3 $$B({\lambda }_{0},\ldots ,{\lambda }_{n})\,:=[\begin{array}{ccccccc}0 & {\lambda }_{0}^{-1/2} & 0 & 0 & \ldots  & 0 & 0\\ {\lambda }_{0}^{-1/2} & 0 & {\lambda }_{1}^{-1/2} & 0 & \ldots  & 0 & 0\\ 0 & {\lambda }_{1}^{-1/2} & 0 & {\lambda }_{2}^{-1/2} & \ldots  & 0 & 0\\  &  &  & \vdots  &  &  & \\ 0 & 0 & 0 & \ldots  & {\lambda }_{n-1}^{-1/2} & 0 & {\lambda }_{n}^{-1/2}\\ 0 & 0 & 0 & \ldots  & 0 & {\lambda }_{n}^{-1/2} & 0\end{array}].$$

Note that *B* is componentwise non-negative and irreducible, so it admits a Perron root *μ* > 0. It has been shown in ref. 46 that *μ* = *R* ^−1/2^. This provides a way to compute the steady-state *R* in the RFM without simulating the dynamical equations of the RFM.

For more on the analysis of the RFM using tools from systems and control theory and the biological implications of this analysis, see refs 46–51. Recently, a network of RFMs, interconnected via a pool of “free” ribosomes, has been used to model and analyze competition for ribosomes in the cell^52^.

## Ribosome Flow Model on a Ring (RFMR)

**Figure 2:**
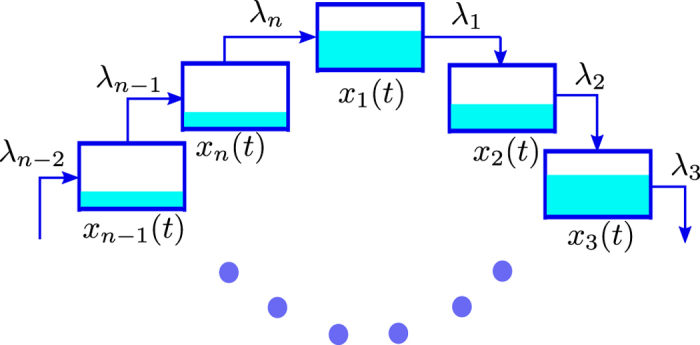
The RFMR models unidirectional flow along a circular chain of *n* sites. The parameter *λ*_*i*_ > 0 controls the transition rate from site *i* to site *i* + 1.

If we consider the RFM under the additional assumption that all the ribosomes leaving site *n* circulate back to site 1 then we obtain the RFMR (see Fig. 2). Just like the RFM, the RFMR is described by *n* nonlinear, first-order ordinary differential equations:

4 $$\begin{array}{rcl}{\dot{x}}_{1} & = & {\lambda }_{n}{x}_{n}(1-{x}_{1})-{\lambda }_{1}{x}_{1}(1-{x}_{2}),\\ {\dot{x}}_{2} & = & {\lambda }_{1}{x}_{1}\mathrm{(1}-{x}_{2})-{\lambda }_{2}{x}_{2}\mathrm{(1}-{x}_{3}),\\  & \vdots  & \\ {\dot{x}}_{n} & = & {\lambda }_{n-1}{x}_{n-1}\mathrm{(1}-{x}_{n})-{\lambda }_{n}{x}_{n}\mathrm{(1}-{x}_{1}\mathrm{)}.\end{array}$$

The difference here with respect to (w.r.t.) the RFM is in the equations describing the change of material in sites 1 and *n*. Specifically, the flow out of site *n* is the flow into site 1. This model assumes perfect recycling (be it covalent or non-covalent), and provides a good approximation when a large fraction of the ribosomes are recycled. Note that the RFMR can also be written succinctly as (1), but now with every index interpreted modulo *n*. In particular, *λ*_0_ [*x*_0_] is replaced by *λ*_*n*_ [*x*_*n*_].

**Remark 1**. It is clear from the cyclic topology of the RFMR that if we cyclically shift all the rates k times for some integer k > 1 then the model does not change.

In the RFMR the sum of the ribosomal densities along the ring at time *t* is given by

$$H(x(t))\,:={x}_{1}(t)+\cdots +{x}_{n}(t).$$

Let *s* denote this value at the initial time *t* = 0, i.e. *s* := *H*(*x*(0)). Since ribosomes that exit site *n* circulate back to site 1, *H*(*t*) is constant for all time, that is, *H*(*x*(*t*)) ≡ *s* for all *t* ≥ 0. The dynamics of the RFMR thus redistributes the particles between the sites, but without changing the sum of densities. In the context of translation, this means that the total number of ribosomes on the (circular) mRNA is conserved. We say that *H*(*x*(*t*)) is a *first integral* of the RFMR.

For *s* ∈ [0, *n*], the *s level set* of *H* is

$${L}_{s}\,:=\{y\in {C}^{n}\,:{y}_{1}+\cdots +{y}_{n}=s\}.$$

This is the set of all possible ribosome density configurations with sum *s*. For example, the vectors of densities $$[\begin{array}{ccccc}s & 0 & 0 & \ldots  & 0\end{array}]^{\prime} $$ and $$[\begin{array}{ccccc}s\mathrm{/2} & s\mathrm{/2} & 0 & \ldots  & 0\end{array}]^{\prime} $$ both belong to *L*_*s*_.

ref. 19 has shown that the RFMR is a strongly cooperative dynamical system, and that this implies that every level set *L*_*s*_ contains a unique steady-state $e=e(s,{\lambda }_{1},\ldots ,{\lambda }_{n})\in {\rm{int}}\,({C}^{n})$, and that a trajectory of the RFMR emanating from any *x*(0) ∈ *L*_*s*_ converges to this steady-state. In particular, the production rate converges to a steady-state value *R* = *R*(*s*, *λ*_1_, …, *λ*_*n*_).

Pick *s* ∈ [0, *n*], and *a* ∈ *L*_*s*_. Consider the RFMR with *x*(0) = *a*. Let

$$\rho \,:=s/n$$

denote the average ribosomal density in the RFMR. At steady-state, i.e. for *x* = *e* the left-hand side of all the equations in (4) is zero, so

5 $$\begin{array}{rcl}{\lambda }_{n}{e}_{n}\mathrm{(1}-{e}_{1}) & = & {\lambda }_{1}{e}_{1}\mathrm{(1}-{e}_{2}),\\  & = & {\lambda }_{2}{e}_{2}\mathrm{(1}-{e}_{3}),\\  & \vdots  & \\  & = & {\lambda }_{n-1}{e}_{n-1}\mathrm{(1}-{e}_{n}),\\  & = & R,\end{array}$$

and, since the sum of densities is conserved,

$${e}_{1}+\cdots +{e}_{n}=s.$$

Note that it follows from (5) that for any *c* > 0

6 $$R(s,c{\lambda }_{1},\ldots ,c{\lambda }_{n})=cR(s,{\lambda }_{1},\ldots ,{\lambda }_{n}),$$

i.e. if we multiply all the rates by a factor *c* > 0 then the steady-state production rate will also increase by the same factor *c*. This implies that the steady-state production rate *R* is *positively homogeneous of order one* w.r.t. the transition rates. There exists an extensive theory of such functions (see e.g ref. 53).

Given a set of transition rates, an interesting question is what ribosomal density *maximizes* the steady-state production rate in the RFMR? Indeed, *s* = 0 means zero production rate (as there are no ribosomes on the ring), and so does *s* = *n*, as all the sites are completely full and the ribosomes cannot move forward. It was shown in ref. 31 that for any arbitrary positive set of rates *λ*_1_, …, *λ*_*n*_, there exists a *unique* sum of densities *s* ^*^ = *s* ^*^(*λ*_1_, …, *λ*_*n*_) (and thus a unique average density *ρ* ^*^ = *s* ^*^/*n*) that maximizes the steady-state production rate. We denote the corresponding optimal steady-state production rate by *R* ^*^ = *R*(*s* ^*^(*λ*_1_, …, *λ*_*n*_), *λ*_1_, …, *λ*_*n*_), and the corresponding optimal steady-state density by *e* ^*^ = *e*(*s* ^*^(*λ*_1_, …, *λ*_*n*_), *λ*_1_, …, *λ*_*n*_). This means that in order to maximize the steady-state production rate (w.r.t. *s*), the mRNA must be initialized with a sum of densities *s* ^*^ (the exact distribution of this sum along the mRNA at time zero is not important). Initializing with either more or less than *s* ^*^ (i.e with ${\sum }_{i=1}^{n}{x}_{i}(0) > {s}^{\ast }$ or with ${\sum }_{i=1}^{n}{x}_{i}(0) < {s}^{\ast }$) will decrease the steady-state production rate w.r.t. the one obtained when the circular mRNA is initialized with ${\sum }_{i=1}^{n}{x}_{i}(0)={s}^{\ast }$.

The results in ref. 31 also show that for the optimal value *s* ^*^, the steady-state density satisfies:

7 $$\prod _{i=1}^{n}{e}_{i}^{\ast }=\prod _{i=1}^{n}(1-{e}_{i}^{\ast }).$$

This can be explained as follows. If the sum of densities *s* is too small then the product ${\prod }_{i=1}^{n}{e}_{i}$ is also small and thus ${\prod }_{i=1}^{n}{e}_{i} < {\prod }_{i=1}^{n}(1-{e}_{i})$. This case is not optimal i.e. it does not maximizes *R*, as there are not enough ribosomes on the ring. If the sum of densities *s* is too large then a similar argument yields ${\prod }_{i=1}^{n}{e}_{i} > {\prod }_{i=1}^{n}(1-{e}_{i})$. This case is also not optimal, as there are too many ribosomes on the ring and this leads to “traffic jams” that reduce the production rate. The optimal scenario lies between these two cases and is characterized by (7).

**Figure 3:**
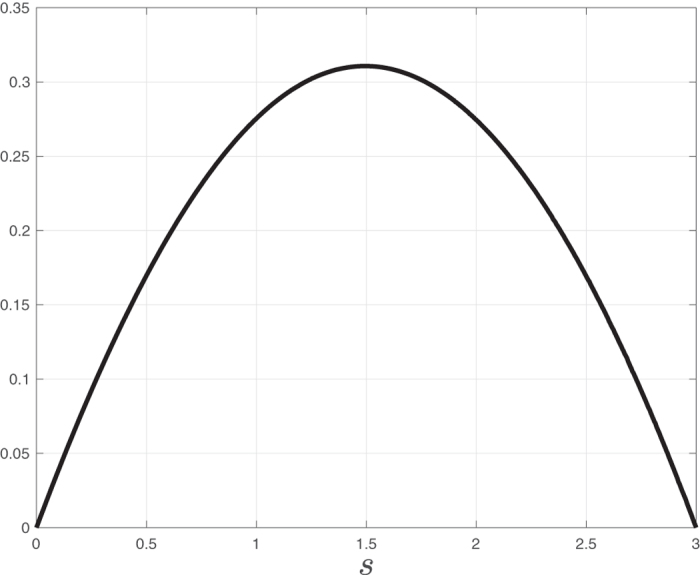
Steady-state production rate *R* as a function of the (conserved) sum of ribosomal densities *s* ∈ [0, 3], for a RFMR with dimension *n* = 3 and transition rates *λ*_1_ = 0.7, *λ*_2_ = 1.6, and *λ*_3_ = 2.2.

**Example 1**. Figure 3 *depicts R as a function of s for a RFMR with dimension n* = 3 *and rates λ*_1_ = 0.7, *λ*_2_ = 1.6, *and λ*_3_ = 2.2. *It may be seen that there exists a unique value s* ^*^ = 1.4948 (*all numerical results in this paper are to four digit accuracy*) *that maximizes R*. *Simulating the RFMR with this initial sum of densities* (*e*.*g*., *by setting* $$x(0)=[\begin{array}{ccc}{s}^{\ast }/2 & 0 & {s}^{\ast }/2\end{array}]^{\prime} $$ *or* $$x(0)=[\begin{array}{ccc}{s}^{\ast }/3 & {s}^{\ast }/3 & {s}^{\ast }/3\end{array}]^{\prime} $$) *yields*

$${e}^{\ast }=[\begin{array}{ccc}0.6878 & 0.3546 & 0.4524\end{array}]^{\prime} ,$$

*and* ${R}^{\ast }={\lambda }_{3}{e}_{3}^{\ast }(1-{e}_{1}^{\ast })=0.3107$. *Note that s* ^*^ *is close* (*but not equal*) *to* 3/2, *that is*, *one half of the maximal possible sum of densities*. *Note also that* ${\prod }_{i=1}^{3}{e}_{i}^{\ast }={\prod }_{i=1}^{3}(1-{e}_{i}^{\ast })=0.1103$.                □

Here, we present for the first time a *spectral representation* of the optimal steady-state production rate *R* ^*^ and the steady-state density *e* ^*^ in the non-homogeneous RFMR. We show that this representation has several advantages. First, it provides an efficient and numerically stable algorithm for evaluating *R* ^*^ and *e* ^*^ (and thus *s* ^*^) even for very large rings. This completely eliminates the need to simulate the RFMR dynamical equations for different values of *s* in order to determine the optimal values. Furthermore, the spectral representation allows to analyze the sensitivity of *R* ^*^ to small changes in the rates. This sensitivity analysis could be crucial for example in synthetic biology applications, where an important problem is to determine positions along the transcript that affect the production rate the most (these are not necessarily the positions of the slowest codons)^54^. Finally, we show that the spectral representation implies that *R* ^*^ is a strictly concave function of the rates. This means that the problem of maximizing *R* ^*^ *w*.*r*.*t*. *the rates* is a convex optimization problem. This problem thus admits a unique solution that can be efficiently determined numerically using algorithms that scale well with *n*.

It is important to note that in general the analysis results for the RFMR hold for any set of transition rates. This is in contrast to the analysis results for TASEP. Rigorous analysis of TASEP seems to be tractable only under the assumption that the internal hopping rates are all equal (i.e. the homogeneous case). In the context of translation, this models the very special case where all elongation rates are assumed to be equal.

The next section derives a spectral representation for *e* ^*^ and *R* ^*^, and describes its implications.

## Main Results

### Spectral Representation

Consider a RFMR with dimension *n* > 2 and rates *λ*_1_, …, *λ*_*n*_ > 0. Define an *n* × *n* matrix

8 $$A({\lambda }_{1},\ldots ,{\lambda }_{n})\,:=[\begin{array}{ccccccc}0 & {\lambda }_{1}^{-1/2} & 0 & 0 & \ldots  & 0 & {\lambda }_{n}^{-1/2}\\ {\lambda }_{1}^{-1/2} & 0 & {\lambda }_{2}^{-1/2} & 0 & \ldots  & 0 & 0\\ 0 & {\lambda }_{2}^{-1/2} & 0 & {\lambda }_{3}^{-1/2} & \ldots  & 0 & 0\\  &  &  & \vdots  &  &  & \\ 0 & 0 & 0 & \ldots  & {\lambda }_{n-2}^{-1/2} & 0 & {\lambda }_{n-1}^{-1/2}\\ {\lambda }_{n}^{-1/2} & 0 & 0 & \ldots  & 0 & {\lambda }_{n-1}^{-1/2} & 0\end{array}].$$

Note that this is a periodic Jacobi matrix (see, e.g ref. 55).

Since *A* is symmetric, all its eigenvalues are real. Since *A* is (componentwise) non-negative and irreducible, it admits a unique maximal eigenvalue *σ* > 0 (called the Perron eigenvalue or Perron root), and a corresponding eigenvector $\zeta \in {{\mathbb{R}}}_{++}^{n}$ (the Perron eigenvector)^56^.

Our first result provides a representation for the optimal steady-state in the RFMR using the spectral properties of the matrix *A*. In what follows, all indexes are interpreted modulo *n*. Recall that all the steady-state properties are invariant to any arbitrary cyclic shifts of the rates (see Remark 1), and that the proofs of all the results are placed in the Appendix.

**Theorem 1**. Consider a RFMR with dimension n and rates λ_1_, …, λ_n_. Let σ > 0 $[\zeta \in {{\mathbb{R}}}_{++}^{n}]$ denote the Perron eigenvalue [eigenvector] of A in (8). Then the optimal values in the RFMR satisfy:

9 $$\begin{array}{rcl}{R}^{\ast } & = & {\sigma }^{-2},\\ {e}_{i}^{\ast } & = & {\lambda }_{i}^{-1/2}{\sigma }^{-1}\frac{{\zeta }_{i+1}}{{\zeta }_{i}},\quad i=1,\ldots ,n,\\ {s}^{\ast } & = & {\sigma }^{-1}\sum _{i=1}^{n}{\lambda }_{i}^{-1/2}\frac{{\zeta }_{i+1}}{{\zeta }_{i}}.\end{array}$$

**Example 2**. *Consider a RFMR with dimension n* = 3 *and rates λ*_1_ = 0.7, *λ*_2_ = 1.6, *and λ*_3_ = 2.2. *The corresponding matrix A is*:

$$A=[\begin{array}{ccc}0 & 1.1952 & 0.6742\\ 1.1952 & 0 & 0.7906\\ 0.6742 & 0.7906 & 0\end{array}].$$

*The maximal eigenvalue of A is σ* = 1.7940, *and the corresponding eigenvector is*

$$\zeta =[\begin{array}{ccc}0.6024 & 0.6219 & 0.5004\end{array}]^{\prime} .$$

*Now* (9) *yields R* ^*^ = 1.7940^−2^ = 0.3107, ${e}_{1}^{\ast }=0.6878$, ${e}_{2}^{\ast }=0.3546$, ${e}_{3}^{\ast }=0.4524$, *and s* ^*^ = 1.4948. *This agrees of course with the results in Example 1*.

Thm. 1 thus provides a spectral representation of the optimal values *R* ^*^, *e* ^*^, and *s* ^*^. One application of this is that the optimal values can be calculated in a numerically stable manner using efficient algorithms for calculating the eigenvalues and eigenvectors of sparse matrices. For a survey of such algorithms see e.g ref. 57. The computation errors are of size *O*(*nε*), where *n* is the dimension of the matrix and *ε* is machine epsilon (approximately 10^−16^ for 64-bit arithmetic). Their time complexity is *O*(*n* ^*c*^) with *c* a constant in the range^2, 3^, implying that they can be applied to very large matrices. For example, for matrices of dimension 10,000 × 10,000 the running times for computing *all* the eigenvalues and eigenvectors are approximately 20 minutes. Since we require only the Perron eigenvalue and eigenvector, better performance is possible using Krylov-subspace eigensolvers, such as ARPACK^58^, that also take advantage of sparsity, and offer to compute small, user-selected subsets of the spectrum.

Thm. 1 has several other interesting implications. Given a RFMR with rates *λ*_1_, …, *λ*_*n*_, define a vector $\bar{\lambda }\in {{\mathbb{R}}}_{++}^{n}$ by ${\bar{\lambda }}_{1}:={\lambda }_{2}$, ${\bar{\lambda }}_{2}:={\lambda }_{3},\ldots ,{\bar{\lambda }}_{n}={\lambda }_{1}$. In other words, $\bar{\lambda }$ is a 1-step cyclic shift of *λ*. Let $P\in {{\mathbb{R}}}^{n\times n}$ be a matrix of zeros, except for the super-diagonal and the (*n*, 1) entry that are all equal to 1. For example, for *n* = 4,

$$P=[\begin{array}{cccc}0 & 1 & 0 & 0\\ 0 & 0 & 1 & 0\\ 0 & 0 & 0 & 1\\ 1 & 0 & 0 & 0\end{array}].$$

Then *P* is a permutation matrix so that *P*′ = *P* ^−1^, and $\bar{\lambda }=P\lambda$. It is straightforward to show that $A(\bar{\lambda })=PA(\lambda )P^{\prime}$, so *A*(*λ*) and $A(\bar{\lambda })$ have the same spectral properties. Thus, Thm. 1 leads to the same steady-state results for both the original RFMR and its cyclic shift and this agrees with Remark 1.

In some special cases, the Perron eigenvalue and eigenvector of *A* may be known explicitly and then one can immediately determine the optimal steady-state in the corresponding RFMR. The next example demonstrates this.

**Example 3**. Consider a RFMR with homogeneous transition rates, i.e.

10 $${\lambda }_{1}=\cdots ={\lambda }_{n}\,:={\lambda }_{c},$$

*where λ*_*c*_ *denotes the common value of all the rates*. *Then it is straightforward to verify that A*(*λ*_*c*_, …, *λ*_*c*_) *admits a Perron eigenvalue* $\sigma =2{\lambda }_{c}^{-1/2}$ *and a corresponding eigenvector* $$\zeta =[\begin{array}{cccc}1 & 1 & \cdots  & 1\end{array}]^{\prime} $$. *Thm*. *1 implies that R* ^*^ = *λ*_*c*_/4 *and* ${e}_{i}^{\ast }=1/2$, *i* = 1, …, *n*. *This result has already been proven in [31*, *Prop*. *3] using a different approach*.      □

### Steady-State RFM as a Special Case of the Optimal Steady-State RFMR

Comparing the spectral representations for the RFMR and the RFM yields the following result. Consider a RFMR with dimension *n*, fixed rates *λ*_1_, …, *λ*_*n*−1_, and *λ*_*n*_ → ∞. In this case, the matrix *A*(*λ*) in (8) converges to the matrix:

11 $$[\begin{array}{ccccccc}0 & {\lambda }_{1}^{-1/2} & 0 & 0 & \ldots  & 0 & 0\\ {\lambda }_{1}^{-1/2} & 0 & {\lambda }_{2}^{-1/2} & 0 & \ldots  & 0 & 0\\ 0 & {\lambda }_{2}^{-1/2} & 0 & {\lambda }_{3}^{-1/2} & \ldots  & 0 & 0\\  &  &  & \vdots  &  &  & \\ 0 & 0 & 0 & \ldots  & {\lambda }_{n-2}^{-1/2} & 0 & {\lambda }_{n-1}^{-1/2}\\ 0 & 0 & 0 & \ldots  & 0 & {\lambda }_{n-1}^{-1/2} & 0\end{array}].$$

Comparing this with (3) and using Thm. 1 imply the following.

**Corollary 1**. Let $${e}^{\ast }=[\begin{array}{ccc}{e}_{1}^{\ast } & \ldots  & {e}_{n}^{\ast }\end{array}]^{\prime} $$ denote the optimal steady-state of a RFMR with dimension n and rates λ_1_, …, λ_n_, where λ_n_ → ∞. Let $$\tilde{e}=[\begin{array}{ccc}{\tilde{e}}_{1} & \ldots  & {\tilde{e}}_{n-2}\end{array}]$$ denote the steady-state of a RFM with dimension n − 2 and transition rates ${\tilde{\lambda }}_{0}={\lambda }_{1},{\tilde{\lambda }}_{1}={\lambda }_{2},\ldots ,{\tilde{\lambda }}_{n-2}={\lambda }_{n-1}$. Then $$\tilde{e}=[\begin{array}{cccc}{e}_{2}^{\ast } & {e}_{3}^{\ast } & \ldots  & {e}_{n-1}^{\ast }\end{array}]^{\prime} $$.

In other words the steady-state of an RFM with arbitrary dimension *m* and arbitrary rates ${\tilde{\lambda }}_{i} > 0$ can be derived from the steady-state of an RFMR with dimension *n*: = *m* + 2, rates ${\lambda }_{i}={\tilde{\lambda }}_{i-1}$, *i* = 1, …, *n* − 1, *λ*_*n*_ → ∞, that is initialized with the optimal sum of densities *s* ^*^. In this respect, the RFM is a kind of “open-boundaries” RFMR that is initialized with the optimal sum of densities.

This connection between the two models can be explained as follows. By (4), in an RFMR with *λ*_*n*_ → ∞, the steady-state density at site *n* will be zero, and at site 1 it will be one. Indeed, the transition rate from site *n* to site 1 is infinite, so site *n* will be completely emptied and site 1 completely filled. This “disconnects” the ring at the link from site *n* to site 1. Furthermore, the completely full site 1 serves as a “source” to site 2 whereas the completely empty site *n* serves as a “sink” to site *n* − 1. The result is that sites 2, …, *n* − 1, of the RFMR become a RFM with dimension *n* − 2. The next example demonstrates this.

**Example 4**. *Consider a RFMR with dimension n* = 5, *and rates λ*_1_ = 0.8, *λ*_2_ = 0.6, *λ*_3_ = 0.4, *λ*_4_ = 0.7, *and λ*_5_ = 0.5. *The optimal steady-state values are*:

$${e}^{\ast }=[\begin{array}{ccccc}0.4260 & 0.5831 & 0.5939 & 0.4019 & 0.4950\end{array}]^{\prime} ,{R}^{\ast }=0.1421.$$

*For λ*_5_ = 100, *the optimal steady-state values are*:

$${e}^{\ast }=[\begin{array}{ccccc}0.9440 & 0.7628 & 0.6087 & 0.2643 & 0.0320\end{array}]^{\prime} ,{R}^{\ast }=0.1791,$$

*for λ*_5_ = 10,000, *the optimal steady-state values are*:

$${e}^{\ast }=[\begin{array}{ccccc}0.9942 & 0.7727 & 0.6100 & 0.2591 & 0.0031\end{array}]^{\prime} ,{R}^{\ast }=0.1808,$$

*and for λ*_5_ = 1,000,000, *they are*:

12 $${e}^{\ast }=[\begin{array}{ccccc}0.9994 & 0.7737 & 0.6102 & 0.2586 & 0.0003\end{array}]^{\prime} ,{R}^{\ast }=0.1810.$$

*It may be observed that as λ*_5_ *increases*, *the optimal steady-state density at site* 5 *[site* 1*] decreases [increases] to zero [one]*. *On the other hand*, *for a RFM with dimension n* = 3 *and rates* ${\tilde{\lambda }}_{0}=\mathrm{0.8,}{\tilde{\lambda }}_{1}=\mathrm{0.6,}{\tilde{\lambda }}_{2}=0.4$, *and* ${\tilde{\lambda }}_{3}\mathrm{=0.7}$, *the steady-state values are:* $$\tilde{e}=[\begin{array}{ccc}0.7738 & 0.6102 & 0.2585\end{array}]^{\prime} $$, *and* $\tilde{R}=0.1810$ (*compare to* (12)).        □

### Sensitivity Analysis

Recall that given the transition rates *λ*_1_, …, *λ*_*n*_, the RFMR admits a unique sum of densities *s* ^*^(*λ*_1_, …, *λ*_*n*_) for which the steady-state production rate is maximized. Maximizing the steady-state production rate is a standard goal in biotechnology, and since codons may be replaced by their synonyms, an important question in the context of the RFMR is: how will a change in the rates affect the maximal production rate *R* ^*^? Note that the effect here is compound, as changing the rates also changes the optimal sum of densities that yields the maximal production rate.

In this section, we analyze

13 $${\varphi }_{i}({\lambda }_{1},\ldots ,{\lambda }_{n})\,:=\frac{\partial }{\partial {\lambda }_{i}}{R}^{\ast }({\lambda }_{1},\ldots ,{\lambda }_{n}),\quad i=1,\ldots ,n,$$

i.e. the sensitivity of the optimal steady-state production rate *R* ^*^ w.r.t. *λ*_*i*_.

A relatively large value of *ϕ*_*i*_ indicates that a small change in *λ*_*i*_ will have a strong impact on the optimal steady-state production rate *R* ^*^. In other words, the sensitivities indicate which rates are the most “important” in terms of their effect on *R* ^*^. The results in Thm. 1 allow to compute the sensitivities using the spectral properties of the matrix *A*.

**Proposition 1**. The sensitivities satisfy:

14 $${\varphi }_{i}=\frac{2{\zeta }_{i}{\zeta }_{i+1}}{{\sigma }^{3}{\lambda }_{i}^{3/2}\zeta ^{\prime} \zeta },\quad i=1,\ldots ,n.$$

Equation (14) provides an efficient and numerically stable method to calculate the sensitivities for large-scale rings and arbitrary positive rates *λ*_*i*_s using standard algorithms for computing the eigenvalues and eigenvectors of periodic Jacobi matrices. Note that (14) implies that all the sensitivities are positive.

**Figure 4:**
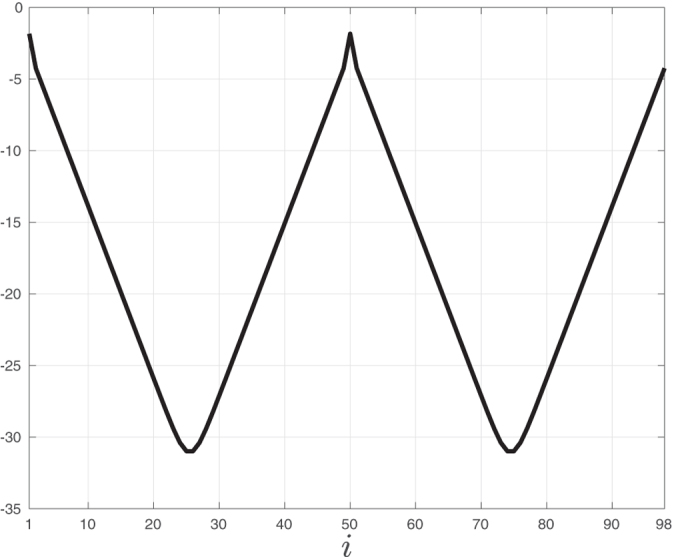
*ln*(*ϕ*_*i*_) as a function of *i* for a RFMR with *n* = 98 and with rates *λ*_1_ = *λ*_50_ = 0.3 and *λ*_*i*_ = 1, for all other rates. Note that the maximal sensitivities are *ϕ*_1_, *ϕ*_50_, and that the sensitivities decrease as we move away from sites 1 and 50 (recall that the topology is circular).

**Example 5**. Figure 4 *depicts ln*(*ϕ*_*i*_), *computed using* (14), *as a function of i for a RFMR with dimension n* = 98 *and rates λ*_1_ = *λ*_50_ = 0.3 *and λ*_*i*_ = 1 *for all other i*. *Here the maximal sensitivity is ϕ*_1_ = *ϕ*_50_, *and the sensitivities decrease as we move away from sites* 1 *and* 50. *This makes sense as the corresponding rates are the bottleneck rates in this example*.

Equation (14) implies that

15 $$\frac{{\varphi }_{i}}{{\varphi }_{j}}=\frac{{\zeta }_{i}{\zeta }_{i+1}}{{\zeta }_{j}{\zeta }_{j+1}}{(\frac{{\lambda }_{j}}{{\lambda }_{i}})}^{3/2},\quad i,j\in \{1,\ldots ,n\},$$

that is, the ratio between any two sensitivities is determined by the corresponding Perron eigenvalue components and the corresponding rates. One may expect that the highest sensitivity will correspond to the minimal rate, but (15) shows that this is not necessarily so. The next example demonstrates this.

**Example 6**. Consider a RFMR with dimension n = 7 and rates:

$$\lambda =[\begin{array}{ccccccc}1 & 1.1 & 0.55 & 1.4 & 1.3 & 0.95 & 0.6\end{array}]^{\prime} .$$

*In this case*, *R* ^*^ = 0.2213. *Using* (*14*) *yields the sensitivities*:

$$\varphi =[\begin{array}{ccccccc}0.0355 & 0.0288 & 0.0774 & 0.0129 & 0.0124 & 0.0298 & 0.0820\end{array}]^{\prime} .$$

*Note that although the minimum rate is λ*_3_, *the maximal sensitivity is ϕ*_7_. *This implies that increasing λ*_7_ *by some small value ε* > 0 *will increase R* ^*^ *more than the increase due to increasing any other rate by ε*. *For example*, *increasing λ*_3_ *by* 0.05 (*and leaving all other rates unchanged*) *yields R* ^*^ = 0.2248, *while increasing λ*_7_ *by* 0.05 *instead* (*and leaving all other rates unchanged*) *yields R* ^*^ = 0.2251.

The spectral approach can also be used to derive theoretical results on the sensitivities. The next three results demonstrate this.

**Proposition 2**. *The sensitivities satisfy* 0 < *ϕ*_*i*_ ≤ 1 *for all i* = 1, …, *n*.

This implies that an increase [decrease] in any of the rates by *ε* increases [decreases] the optimal steady-state production rate by no more than *ε*.

**Proposition 3**. Consider a RFMR with dimension n and homogeneous rates (10). Then

$${\varphi }_{i}=\frac{1}{4n},\quad i=1,\ldots ,n.$$

This means that in the homogeneous case, all the sensitivities are equal. This is of course expected, as the circular topology of the sites implies that all the rates have the same effect on *R* ^*^. Furthermore, the sensitivities decrease with *n*, i.e. in a longer ring each rate has a smaller effect on *R* ^*^.

Assume now that the RFMR rates satisfy

16 $${\lambda }_{i}={\lambda }_{n-i},\quad i=1,\ldots ,n-1,$$

i.e. the rates are *symmetric*. Note that since all indexes are interpreted modulo *n*, it is enough that (16) holds for some cyclic permutation of the rates. For example, for *n* = 3 the rates are symmetric if at least two of the rates *λ*_1_, *λ*_2_, *λ*_3_ are equal.

**Proposition 4**. Consider a RFMR with dimension n and symmetric rates (16). Then

$${\varphi }_{i}={\varphi }_{n-i},\quad i=1,\ldots ,n-1.$$

In other words, the symmetry of the rates implies symmetry of the sensitivities.

**Example 7**. *Consider a RFMR with dimension n* = 6 *and rates λ*_1_ = *λ*_5_ = 1, *λ*_2_ = *λ*_4_ = 1.2, *λ*_3_ = 0.8 *and λ*_6_ = 1.5. *Note that these rates satisfy* (16). *The sensitivities are*:

$$\varphi =[\begin{array}{cccccc}0.0408 & 0.0388 & 0.0804 & 0.0388 & 0.0408 & 0.0200\end{array}]^{\prime} ,$$

*and it may be observed that ϕ*_*i*_ = *ϕ*_6−*i*_, *i* = 1, …, 5.                          □

### Optimizing the Production Rate

Any set of rates *λ* = (*λ*_1_, …, *λ*_*n*_) induces an optimal sum of densities *s* ^*^, and the RFMR initialized with this sum of densities yields a maximal production rate *R* ^*^ (w.r.t. all other initial conditions). This yields a mapping *λ* → *R* ^*^(*λ*). Now suppose that we have some compact set, denoted by Ω, of *n*-dimensional vectors with positive entries. Every vector from Ω can be used as a set of rates *λ* for the RFMR, and thus yields a value *R* = *R* ^*^(*λ*).

A natural problem is finding a vector in Ω that yields the maximal value of *R* ^*^ over all vectors in Ω. We denote a vector in Ω that yields the maximal *R* ^*^ by *η*, i.e.

$$\eta \,:=\mathop{{\rm{\arg }}\,{\rm{\max }}}\limits_{\lambda \in {\rm{\Omega }}}{R}^{\ast }(\lambda \mathrm{)}.$$

In the context of translation, this means that a circular mRNA with rates *η*, initialized with *s* ^*^(*η*), will yield a steady-state production rate that is larger than that obtained for all the other options for the rate vector in Ω (regardless of the initial sum of densities in these other circular mRNAs).

The next result is useful for efficiently analyzing the maximization of *R* ^*^ w.r.t. the rates.

**Proposition 5**. Consider a RFMR with dimension n. The mapping $\lambda \,:=({\lambda }_{1},\ldots ,{\lambda }_{n})\mapsto {R}^{\ast }(\lambda )$ is strictly concave on ${{\mathbb{R}}}_{++}^{n}$.

For example, for *n* = 2 it is straightforward to show that

$${R}^{\ast }({\lambda }_{1},{\lambda }_{2})=\frac{{\lambda }_{1}{\lambda }_{2}}{{(\sqrt{{\lambda }_{1}}+\sqrt{{\lambda }_{2}})}^{2}}.$$

**Figure 5:**
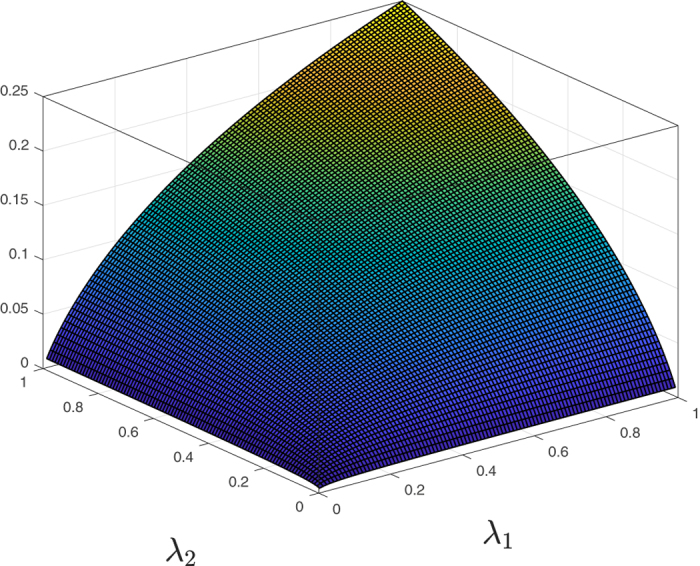
*R* ^*^(*λ*_1_, *λ*_2_) in RFMR with *n* = 2 as a function of its parameters.

Figure 5 depicts *R* ^*^(*λ*_1_, *λ*_2_) as a function of its parameters. It may be observed that this is a strictly concave function on ${{\mathbb{R}}}_{++}^{2}$.

The sensitivity analysis of *R* ^*^, and its strict concavity w.r.t. the rates, have important implications to the problem of optimizing the steady-state production rate in the RFMR w.r.t. the rates *λ*. We now explain this using a specific optimization problem. First note that to make the problem meaningful every rate must be bounded from above. Otherwise, the optimal solution will be to take this rate to infinity. We thus consider the following constrained optimization problem.

**Problem 1**. Consider a RFMR with dimension n. Given the parameters w_1_, …, w_n_, b > 0, maximize R^*^ = R^*^(λ_1_, …, λ_n_) w.r.t. the parameters λ_1_, …, λ_n_, subject to the constraints

17 $$\begin{array}{c}\sum _{i=1}^{n}{w}_{i}{\lambda }_{i}\le b,\\ {\lambda }_{1},\ldots ,{\lambda }_{n} > 0.\end{array}$$

In other words, the problem is to maximize *R* ^*^ w.r.t. the rates, under the constraints that the rates are positive and their weighted sum is bounded by *b*. The weights *w*_*i*_ can be used to provide different weighting to the different rates, and *b* represents the “total biocellular budget”. By Prop. 2, the optimal solution always satisfies the constraint in (17) with equality. Note that a similar optimization problem was defined and analyzed in the context of the RFM in ref. 46.

In the context of mRNA translation, each *λ*_*i*_ depends on the availability of translation resources that affect codon decoding times, such as tRNA molecules, amino acids, elongation factors, and Aminoacyl tRNA synthetases. These resources are limited as generating them consumes significant amounts of cellular energy. They are also correlated. For example, a large *λ*_*i*_ may imply large consumption of certain tRNA molecules by site *i*, depleting the availability of tRNA molecules to the other sites. Thus, the first (affine) constraint in (17) describes the limited and shared translation resources, whereas *b* describes the total available biocellular budget.

By Prop. 5, the objective function in Problem 1 is strictly concave, and since the constraints are affine, Problem 1 is a *convex optimization problem* ^59^. Thus, it admits a *unique* solution. We denote the optimal solution of Problem 1 by ${\lambda }^{co}\,:=({\lambda }_{1}^{co},\ldots ,{\lambda }_{n}^{co})$, and the corresponding maximal (now in the sense of both transition rates and sum of densities) steady-state production rate by *R* ^*co*^ (where *co* denotes constrained optimization). This means that for a RFMR with dimension *n*, *R* ^*co*^ is the maximal steady-state production rate over all the rates satisfying the constraints (17) and all possible initial densities.

The convexity also implies that the solution can be determined efficiently using numerical algorithms that scale well with *n*. To demonstrate this, we wrote a simple and *unoptimized* MATLAB program (that is guaranteed to converge because of the convexity) for solving this optimization problem and ran it on a MAC laptop with a 2.6 GHz Intel core i7 processor. As an example, for *n* = 100 and the (arbitrarily chosen) weights *w*_*i*_ = 1 + 0.4sin(2*πi*/100), *i* = 1, …, 100, and *b* = 1, the optimal solution was found after 11.7 seconds.

The affine constraint in (17) includes a possibly different weight for each of the rates. For example, if *w*_2_ is much larger than the other weights then this means that any small increase in *λ*_2_ will greatly increase the total weighted sum, thus typically forcing the optimal value ${\lambda }_{2}^{co}$ to be small. In the special case where all the *w*_*i*_s are equal the formulation gives equal preference to all the rates, so if the corresponding optimal solution satisfies ${\lambda }_{j}^{co} > {\lambda }_{i}^{co}$, for some *i*, *j*, then this implies that, in the context of maximizing *R* ^*^, *λ*_*j*_ is “more important” than *λ*_*i*_. We refer to this case as the *homogeneous constraint* case and assume, without loss of generality, that *w*_*i*_ = 1 for all *i*. Note that by (6) we can always assume, without loss of generality, that *b* = 1.

**Proposition 6**. *Consider Problem 1 with* ${w}_{1}=\cdots ={w}_{n}=b=1$, *i*.*e*. *the affine constraint is*

18 $$\sum _{i=1}^{n}{\lambda }_{i}=1.$$

*Then the optimal solution is* ${\lambda }_{i}^{co}=1/n$ *for all i*. *The RFMR with these rates satisfies s* ^*^ = *n*/2, ${e}_{i}^{co}=1/2$ *for all i*, *and R* ^*co*^ = 1/(4*n*).

**Remark 2**. *In view of the Kuhn–Tucker theorem* ^59^, *the necessary and sufficient condition for optimality of λ in Problem 1 with homogeneous weights is that the sensitivity* ${\varphi }_{i}=\frac{\partial {R}^{\ast }}{\partial {\lambda }_{i}}({\lambda }^{\ast })$ *does not depend on the index i*.

## Discussion

We considered a deterministic model for translation along a circular mRNA. The behavior of this model depends on the transition rates between the sites and on the value $s\,:={\sum }_{i=1}^{n}{x}_{i}(0)$, that is, the sum of densities along the ring at the initial time *t* = 0. The sum of densities is conserved, so ${\sum }_{i=1}^{n}{x}_{i}(t)=s$ for all *t* ≥ 0.

We derived a spectral representation for the steady-state density and production rate for the case where the initial sum of densities is the unique value *s* ^*^, i.e. the sum yielding a maximal steady-state production rate. In fact, the proof of Thm. 1 (see the Appendix) shows that we can interpret the optimal density RFMR as a dynamical system that “finds” the Perron eigenvalue and eigenvector of a certain periodic Jacobi matrix.

The spectral representation for the RFMR provides a powerful framework for analyzing the RFMR when initialized with the optimall sum of densities *s* ^*^. In addition to providing an efficient and numerically stable manner for computing the optimal steady-state production rate and steady-state density, it allows to efficiently compute the sensitivity of the optimal steady-state production rate to perturbations in the transition rates. This is important as conditions in the cell are inherently stochastic, and thus sensitivity analysis must accompany the steady-state description.

Furthermore, using the spectral representation, it was shown that the steady-state production rate with optimal sum of densities is a strictly concave function of the RFMR rates. The translation machinery in the cell is affected by different kinds of mutations (e.g. synonymous codon mutations, duplication of a tRNA gene, etc.). The strict concavity result thus suggests that (at least for highly expressed genes, like ribosomal proteins) the selection of mutations that increase fitness may indeed converge towards the unique optimal parameter values by a simple “hill-climbing” evolution process. The strict concavity implies that given an affine (and more generally convex) constraint on the rates, that represents limited and shared translation resources, the unique optimal set of rates can be determined efficiently even for (circular) mRNAs with a large number of codons.

Obtaining an optimal production rate is an important problem in synthetic biology and biotechnology. Examples include optimal synonymous codon mutations of an endogenous gene, and optimal translation efficiency and protein levels of heterologous genes in a new host^39, 60–62^. These genes compete with endogenous genes for the available translation resources, as consuming too much resources by the heterologous gene may kill the host^60, 61^. Thus, in scenarios where the relevant resources are scarce and survival of the cell is important any realistic optimization of the protein production rate should explicitly limit resource consumption, as otherwise the fitness of the host may be significantly reduced. The constrained optimization problem presented and analyzed here may thus be a useful tool in certain synthetic biology and biotechnology applications.

We also showed that the spectral representation of the RFM follows as a special case of the representation given here for the RFMR. However, a better understanding of the link between the RFM and the RFMR requires further study. Our results suggest several directions for future research. One such direction is finding special cases, besides the one described in Example 2.1, where the Perron eigenvalue and eigenvector of *A*(*λ*_1_, …, *λ*_*n*_) are explicitly known. Another possible direction is the analysis of the dual of the optimization problem defined by Problem 1. Specifically, does the dual problem have any interesting biological interpretation in the context of translation, and does its analysis provide more insight into optimizing translation?

Finally, TASEP with periodic boundary conditions has been used to model many transport phenomena including traffic flow and pedestrian dynamics^16, 63^. We believe that the spectral representation of the RFMR with optimal sum of densities may be useful also for analyzing other applications.

### Data availability statement

All the relevant data is included in the manuscript.

## Appendix - Proofs

**Proof of Thm. 1**. Pick *n* > 2 and parameters *c*_1_, …, *c*_*n*−1_ > 0, and *c*_*n*_ ≥ 0. Consider the *n* × *n* periodic Jacobi matrix:

$$J\,:=[\begin{array}{ccccccccc}0 & {c}_{1} & 0 & 0 & \ldots  & 0 & 0 & 0 & {c}_{n}\\ {c}_{1} & 0 & {c}_{2} & 0 & \ldots  & 0 & 0 & 0 & 0\\ 0 & {c}_{2} & 0 & {c}_{3} & \ldots  & 0 & 0 & 0 & 0\\  &  &  &  & \vdots  &  &  &  & \\ 0 & 0 & 0 & 0 & \ldots  & 0 & {c}_{n-2} & 0 & {c}_{n-1}\\ {c}_{n} & 0 & 0 & 0 & \ldots  & 0 & 0 & {c}_{n-1} & 0\end{array}].$$

Note that *J* is irreducible and (componentwise) non-negative. Let *σ* > 0 denote that Perron eigenvalue of *J* and let $\zeta \in {{\mathbb{R}}}_{++}^{n}$ denote the corresponding eigenvector. The equation *Jζ* = *σζ* yields

19 $$\begin{array}{rcl}{c}_{1}{\zeta }_{2}+{c}_{n}{\zeta }_{n} & = & \sigma {\zeta }_{1},\\ {c}_{1}{\zeta }_{1}+{c}_{2}{\zeta }_{3} & = & \sigma {\zeta }_{2},\\ {c}_{2}{\zeta }_{2}+{c}_{3}{\zeta }_{4} & = & \sigma {\zeta }_{3},\\  & \vdots  & \\ {c}_{n-2}{\zeta }_{n-2}+{c}_{n-1}{\zeta }_{n} & = & \sigma {\zeta }_{n-1},\\ {c}_{n}{\zeta }_{1}+{c}_{n-1}{\zeta }_{n-1} & = & \sigma {\zeta }_{n}.\end{array}$$

Define

20 $${d}_{i}:=\frac{{c}_{i}{\zeta }_{i+1}}{\sigma {\zeta }_{i}},\quad i=1,\ldots ,n.$$

Note that since the indexes are interpreted modulo *n*, Eq. (20) implies in particular that

21 $${d}_{n}=\frac{{c}_{n}{\zeta }_{1}}{\sigma {\zeta }_{n}}.$$

Then (19) yields:

22 $$\begin{array}{rcl}{\sigma }^{-2} & = & {c}_{n}^{-2}{d}_{n}(1-{d}_{1}),\\ {\sigma }^{-2} & = & {c}_{1}^{-2}{d}_{1}(1-{d}_{2}),\\ {\sigma }^{-2} & = & {c}_{2}^{-2}{d}_{2}(1-{d}_{3}),\\  & \vdots  & \\ {\sigma }^{-2} & = & {c}_{n-2}^{-2}{d}_{n-2}(1-{d}_{n-1}),\\ {\sigma }^{-2} & = & {c}_{n-1}^{-2}{d}_{n-1}(1-{d}_{n}).\end{array}$$

Also, it follows from (20) that ${\prod }_{i=1}^{n}{d}_{i}={\sigma }^{-n}{\prod }_{i=1}^{n}{c}_{i}$, and from (22) that ${\prod }_{i=1}^{n}(1-{d}_{i})={\sigma }^{-2n}\frac{{\prod }_{i=1}^{n}{c}_{i}^{2}}{{\prod }_{i=1}^{n}{d}_{i}}$, and combining these two equations yields

23 $$\prod _{i=1}^{n}{d}_{i}=\prod _{i=1}^{n}(1-{d}_{i}).$$

Note that all the derivations above hold for any real eigenvalue of *J* and its corresponding eigenvector (assuming all its entries are non zero so that (20) is well-defined), but since the Perron eigenvector is the only eigenvector in the first orthant^56^, all the *d*_*i*_s are positive only for the Perron eigenvalue and eigenvector.

Now consider a RFMR with dimension *n* and rates ${\lambda }_{i}\,:={c}_{i}^{-2}$, *i* = 1, …, *n*, that is:

24 $$\begin{array}{rcl}{\dot{x}}_{1} & = & {c}_{n}^{-2}{x}_{n}(1-{x}_{1})-{c}_{1}^{-2}{x}_{1}(1-{x}_{2})\\ {\dot{x}}_{2} & = & {c}_{1}^{-2}{x}_{1}(1-{x}_{2})-{c}_{2}^{-2}{x}_{2}(1-{x}_{3})\\  & \vdots  & \\ {\dot{x}}_{n-1} & = & {c}_{n-2}^{-2}{x}_{n-2}(1-{x}_{n-1})-{c}_{n-1}^{-2}{x}_{n-1}(1-{x}_{n})\\ {\dot{x}}_{n} & = & {c}_{n-1}^{-2}{x}_{n-1}(1-{x}_{n})-{c}_{n}^{-2}{x}_{n}(1-{x}_{1}).\end{array}$$

We already know that this system converges to a steady-state *e* ∈ *C* ^*n*^, that is,

$$R={c}_{n}^{-2}{e}_{n}(1-{e}_{1})={c}_{1}^{-2}{e}_{1}(1-{e}_{2})=\cdots ={c}_{n-1}^{-2}{e}_{n-1}(1-{e}_{n}).$$

Comparing this with (22) shows that *e*_*i*_ = *d*_*i*_ for all *i*, and that the steady-state production rate is *R* = *σ* ^−2^. Furthermore, (23) implies that ${\prod }_{i\mathrm{=1}}^{n}{e}_{i}={\prod }_{i\mathrm{=1}}^{n}\mathrm{(1}-{e}_{i})$, so we conclude that the steady-state satisfies condition (7) that describes the unique optimal steady-state (i.e. the steady-state production rate that corresponds to the unique optimal sum of densities *s* ^*^). This proves the first two equations in (9). Finally, since the sum of densities is conserved, it is equal to ${\sum }_{i\mathrm{=1}}^{n}{e}_{i}$. This completes the proof of Thm. 1.                    □

**Proof of Proposition 1**. By Thm. 1,

25 $${\varphi }_{i}=\frac{\partial }{\partial {\lambda }_{i}}{\sigma }^{-2}=-2{\sigma }^{-3}\frac{\partial \sigma }{\partial {\lambda }_{i}}.$$

By known results from linear algebra (see, e.g. ref. 64), the sensitivity of the Perron root of *A* w.r.t. a change in *λ*_*i*_ is

$$\frac{\partial }{\partial {\lambda }_{i}}\sigma =\frac{\zeta ^{\prime} (\frac{d}{d{\lambda }_{i}}A)\zeta }{\zeta ^{\prime} \zeta }.$$

Only the entries ${a}_{i,i+1}={a}_{i+1,i}={\lambda }_{i}^{-1/2}$ depend on *λ*_*i*_, so

$$\frac{\partial }{\partial {\lambda }_{i}}\sigma =\frac{-{\zeta }_{i}{\zeta }_{i+1}{\lambda }_{i}^{-3/2}}{\zeta ^{\prime} \zeta },$$

and combining this with (25) proves (14).                             □

**Proof of Prop. 2**. Since *σ* > 0 and $\zeta \in {{\mathbb{R}}}_{++}^{n}$, *ϕ*_*i*_ > 0 for all *i*. To prove the upper bound, perturb *λ*_*i*_ to ${\bar{\lambda }}_{i}:={\lambda }_{i}+\varepsilon$, with *ε* > 0 sufficiently small. This yields a perturbed matrix $\bar{A}$ that is identical to *A* except for entries (*i*, *i* + 1) and (*i* + 1, *i*) that are

$${\bar{\lambda }}_{i}^{-1/2}={({\lambda }_{i}+\varepsilon )}^{-1/2}={\lambda }_{i}^{-1/2}-\frac{\varepsilon {\lambda }_{i}^{-3/2}}{2}+o(\varepsilon ),$$

where *o*(*ε*) denotes a function *f*(*ε*) satisfying ${\mathrm{lim}}_{\varepsilon \to 0}\frac{f(\varepsilon )}{\varepsilon }=0$. This means that $\bar{A}=A+P$, where $P\in {{\mathbb{R}}}^{n\times n}$ is a matrix with zero entries except for entries (*i*, *i* + 1) and (*i* + 1, *i*) that are equal to $-\frac{\varepsilon {\lambda }_{i}^{-3/2}}{2}+o(\varepsilon )$. By Weyl’s inequality^56^, $\rho (\bar{A})\ge \rho (A)-\frac{\varepsilon {\lambda }_{i}^{-3/2}}{2}+o(\varepsilon )$, where *ρ*(*Q*) denotes the maximal eigenvalue of a symmetric matrix *Q*. This means that $\frac{\partial \rho (A)}{\partial {\lambda }_{i}}\ge -\frac{{\lambda }_{i}^{-3/2}}{2}+\frac{o(\varepsilon )}{\varepsilon }$, thus *ϕ*_*i*_ ≤ (*R* ^*^/*λ*_*i*_)^3/2^. Since *R* ^*^ ≤ *λ*_*i*_, it follows that *ϕ*_*i*_ ≤ 1 for all *i*.    □

**Proof of Prop. 3**. Consider a RFMR with homogeneous rates (10). Then by Example 2.1, *ζ*_*i*_ = 1, *i* = 1, …, *n*, and $\sigma =2{\lambda }_{c}^{-\mathrm{1/2}}$, and plugging these in (14) completes the proof.                     □

**Proof of Prop. 4**. We require the following result.

**Proposition 7**. Consider the RFMR with dimension n and symmetric rates. Then ζ_i_ = ζ_n+1−i_, i = 1, …, n.

**Proof of Prop.7**. Consider first the case *n* even. Let $Q\in {{\mathbb{R}}}^{(n\mathrm{/2)}\times (n\mathrm{/2)}}$ be a reversal matrix, i.e. a matrix of zeros except for the counter-diagonal (i.e. entries $(i,\frac{n}{2}-i+1)$, *i* = 1, …, *n*/2) that is all ones. For example, for *n* = 4,

$$Q=[\begin{array}{cc}0 & 1\\ 1 & 0\end{array}].$$

Note that given any arbitrary vector $$v=[\begin{array}{cccc}{v}_{1} & {v}_{2} & \cdots  & {v}_{n/2}\end{array}]^{\prime} \in {{\mathbb{R}}}^{n\mathrm{/2}}$$, $$Qv=[\begin{array}{cccc}{v}_{n/2} & {v}_{(n/2)-1} & \cdots  & {v}_{1}\end{array}]^{\prime} $$.

Since the rates satisfy (16), the *n* × *n* matrix *A* has the form

$$A=[\begin{array}{cc}{A}_{1} & {A}_{2}\\ Q{A}_{2}Q & Q{A}_{1}Q\end{array}],$$

where ${A}_{1}\in {{\mathbb{R}}}_{+}^{(n\mathrm{/2)}\times (n\mathrm{/2)}}$ is a matrix of zeros except for the super-diagonal and the sub-diagonal, which are both equal to $({\lambda }_{1}^{-1/2},\ldots ,{\lambda }_{(n/2)-1}^{-1/2})$, and ${A}_{2}\in {{\mathbb{R}}}_{+}^{(n\mathrm{/2)}\times (n\mathrm{/2)}}$ is a matrix of zeros except for entry (1, *n*/2) that is ${\lambda }_{n}^{-\mathrm{1/2}}$, and entry (*n*/2, 1) that is ${\lambda }_{n\mathrm{/2}}^{-\mathrm{1/2}}$. Decompose the Perron eigenvector *ζ* of *A* as $${\zeta }^{1}\,:=[\begin{array}{ccc}{\zeta }_{1} & \ldots  & {\zeta }_{n/2}\end{array}]^{\prime} $$ and $${\zeta }^{2}\,:=[\begin{array}{ccc}{\zeta }_{(n/2)+1} & \ldots  & {\zeta }_{n}\end{array}]^{\prime} $$.

Let *ρ*(*W*) denote the spectral radius of a matrix *W*. Since *A*_1_ is a principal submatrix of the (componentwise) nonnegative matrix *A*, *ρ*(*A*_1_) ≤ *ρ*(*A*) (see [56, Ch. 8]). Assume for the moment that *ρ*(*A*_1_) = *ρ*(*A*). Then using the fact that *QQ* = *I*, that is *Q* = *Q* ^−1^, we conclude that $$\rho ([\begin{array}{cc}{A}_{1} & 0\\ 0 & Q{A}_{1}Q\end{array}])=\rho (A)$$. This means that the matrices $$[\begin{array}{cc}{A}_{1} & 0\\ 0 & Q{A}_{1}Q\end{array}]$$ and $$[\begin{array}{cc}{A}_{1} & {A}_{2}\\ Q{A}_{2}Q & Q{A}_{1}Q\end{array}]$$ have the same Perron root, but this contradicts Prop. 2. We conclude that

26 $$\rho ({A}_{1}) < \rho (A)=\sigma .$$

The equation *Aζ* = *σζ* yields

$$\begin{array}{c}{A}_{1}{\zeta }^{1}+{A}_{2}{\zeta }^{2}=\sigma {\zeta }^{1},\\ Q{A}_{2}Q{\zeta }^{1}+Q{A}_{1}Q{\zeta }^{2}=\sigma {\zeta }^{2}.\end{array}$$

Multiplying both sides of the second equation by *Q*, noting that *QQ* = *I*, and rearranging yield

27 $$\begin{array}{rcl}{A}_{1}{\zeta }^{1}+{A}_{2}{\zeta }^{2} & = & \sigma {\zeta }^{1},\\ {A}_{1}Q{\zeta }^{2}+{A}_{2}Q{\zeta }^{1} & = & \sigma Q{\zeta }^{2}.\end{array}$$

Subtracting the second equation from the first and using again the fact that *QQ* = *I* yields

28 $$({A}_{1}-{A}_{2}Q-\sigma I)({\zeta }^{1}-Q{\zeta }^{2})=0.$$

Combining this with (26) and the fact that *A*_2_ *Q* is (componentwise) nonnegative implies that *ζ* ^1^ = *Qζ* ^2^, i.e. *ζ*_*i*_ = *ζ*_*n*+1−*i*_, *i* = 1, …, *n*. This completes the proof for the case *n* even. The proof when *n* is odd is very similar and therefore omitted. □

Now the proof of Prop. 4 follows from combining (14), Thm. 1, and Prop. 7.              □

**Proof of Prop. 5**. The real scalar mapping $c\mapsto {c}^{-1/2}$ is convex on ${{\mathbb{R}}}_{++}^{n}$. This implies that the map $\lambda =({\lambda }_{1},\ldots ,{\lambda }_{n})\mapsto A(\lambda )$, where *A*(*λ*) is given in (8) is convex, that is, the matrix inequality

29 $$\frac{1}{2}(A(\lambda ^{\prime} )+A(\lambda ^{\prime\prime} ))\ge A(\frac{1}{2}(\lambda ^{\prime} +\lambda ^{\prime\prime} ))$$

holds elementwise for any $\lambda ^{\prime} ,\lambda ^{\prime\prime} \in {{\mathbb{R}}}_{++}^{n}$. The Perron–Frobenius theorem implies the corresponding inequality for the Perron eigenvalue^56^

30 $$\frac{1}{2}(\sigma (A(\lambda ^{\prime} ))+\sigma (A(\lambda ^{\prime\prime} )))\ge \sigma (A(\frac{1}{2}(\lambda ^{\prime} +\lambda ^{\prime\prime} ))),$$

where the inequality (30) is strict if *λ*′ ≠ *λ*′′. Thus, $\lambda \mapsto \sigma (\lambda )$ is a strictly convex function. In view of the identity *R* ^*^ = *σ* ^−2^ in (9), it follows that $\lambda \mapsto {R}^{\ast }(\lambda )$ is a strictly concave function.                   □

**Proof of Prop. 6**. We know that Problem 1 admits a unique optimal solution $\tilde{\lambda }$. Consider the cyclic shift ${\bar{\lambda }}_{i}={\tilde{\lambda }}_{i+1}$, *i* = 1, …, *n*, where the indices are taken modulo *n*. Note that ${\sum }_{i=1}^{n}{\bar{\lambda }}_{i}={\sum }_{i=1}^{n}{\tilde{\lambda }}_{i}=1$, so $\bar{\lambda }$ also satisfies the constraint (18). The matrices $A(\bar{\lambda })$ and $A(\tilde{\lambda })$ have the same spectrum. Since the optimal solution is unique, $\overline{\lambda }=\tilde{\lambda }$. We conclude that the optimal transition rates ${\tilde{\lambda }}_{i}$ are all equal, and thus ${\lambda }_{i}^{co}\,:=1/n$, *i* = 1, …, *n*. By Example 2.1, *R* ^*co*^ = 1/(4*n*), and ${e}_{i}^{co}=1/2$, *i* = 1, …, *n*.                        □
